# Present-day thermal and water activity environment of the Mars Sample Return collection

**DOI:** 10.1038/s41598-024-57458-4

**Published:** 2024-03-26

**Authors:** Maria-Paz Zorzano, Germán Martínez, Jouni Polkko, Leslie K. Tamppari, Claire Newman, Hannu Savijärvi, Yulia Goreva, Daniel Viúdez-Moreiras, Tanguy Bertrand, Michael Smith, Elisabeth M. Hausrath, Sandra Siljeström, Kathleen Benison, Tanja Bosak, Andrew D. Czaja, Vinciane Debaille, Christopher D. K. Herd, Lisa Mayhew, Mark A. Sephton, David Shuster, Justin I. Simon, Benjamin Weiss, Nicolas Randazzo, Lucia Mandon, Adrian Brown, Michael H. Hecht, Jesús Martínez-Frías

**Affiliations:** 1 Centro de Astrobiología (CAB), CSIC-INTA, 28850 Torrejón de Ardoz, Madrid, Spain; 2 Lunar and Planetary Institute, Universities Space Research Association, Houston, TX USA; 3 University of Michigan, Ann Arbor, MI USA; 4 Finnish Meteorological Institute, Helsinki, Finland; 5 Jet Propulsion Laboratory, California Institute of Technology, 4800 Oak Grove Dr., Pasadena, CA 91109 USA; 6 Aeolis Research, Chandler, AZ USA; 7 University of Helsinki, Helsinki, Finland; 8 Laboratoire d’Etudes Spatiales et d’Instrumentation en Astrophysique (LESIA), Observatoire de Paris, Université PSL, CNRS, Sorbonne Université, Univ. Paris Diderot, Sorbonne, France; 9 NASA Goddard Space Flight Center, Greenbelt, MD USA; 10 Department of Geoscience, University of Nevada, Las Vegas, NV USA; 11 RISE Research Institutes of Sweden, Stockholm, Sweden; 12 West Virginia University, Morgantown, WV USA; 13 Department of Earth, Atmospheric, and Planetary Sciences, Massachusetts Institute of Technology, Cambridge, MA USA; 14 Department of Geosciences, University of Cincinnati, Cincinnati, OH USA; 15 Laboratoire G-Time, Université Libre de Bruxelles, Brussels, Belgium; 16 Department of Earth and Atmospheric Sciences, University of Alberta, Edmonton, Canada; 17 Department of Geological Sciences, University of Colorado Boulder, Boulder, CO USA; 18 Department of Earth Science and Engineering, Imperial College London, London, UK; 19 University of California, Berkeley, CA USA; 20 Center for Isotope Cosmochemistry and Geochronology, Astromaterials Research and Exploration Science, NASA Johnson Space Center, Houston, TX USA; 21 Division of Geological and Planetary Sciences, California Institute of Technology, Pasadena, CA USA; 22 Plancius Research, Severna Park, MD USA; 23 MIT Haystack Observatory, Westford, MA 01886 USA; 24 Instituto de Geociencias (CSIC-UCM, Madrid, Spain

**Keywords:** Mars sample return, Water activity, Temperature, Habitability, Jezero, Environment, Planetary science, Astrobiology, Atmospheric dynamics

## Abstract

The Mars Sample Return mission intends to retrieve a sealed collection of rocks, regolith, and atmosphere sampled from Jezero Crater, Mars, by the NASA Perseverance rover mission. For all life-related research, it is necessary to evaluate water availability in the samples and on Mars. Within the first Martian year, Perseverance has acquired an estimated total mass of 355 g of rocks and regolith, and 38 μmoles of Martian atmospheric gas. Using in-situ observations acquired by the Perseverance rover, we show that the present-day environmental conditions at Jezero allow for the hydration of sulfates, chlorides, and perchlorates and the occasional formation of frost as well as a diurnal atmospheric-surface water exchange of 0.5–10 g water per m^2^ (assuming a well-mixed atmosphere). At night, when the temperature drops below 190 K, the surface water activity can exceed 0.5, the lowest limit for cell reproduction. During the day, when the temperature is above the cell replication limit of 245 K, water activity is less than 0.02. The environmental conditions at the surface of Jezero Crater, where these samples were acquired, are incompatible with the cell replication limits currently known on Earth.

## Introduction

**Figure 1:**
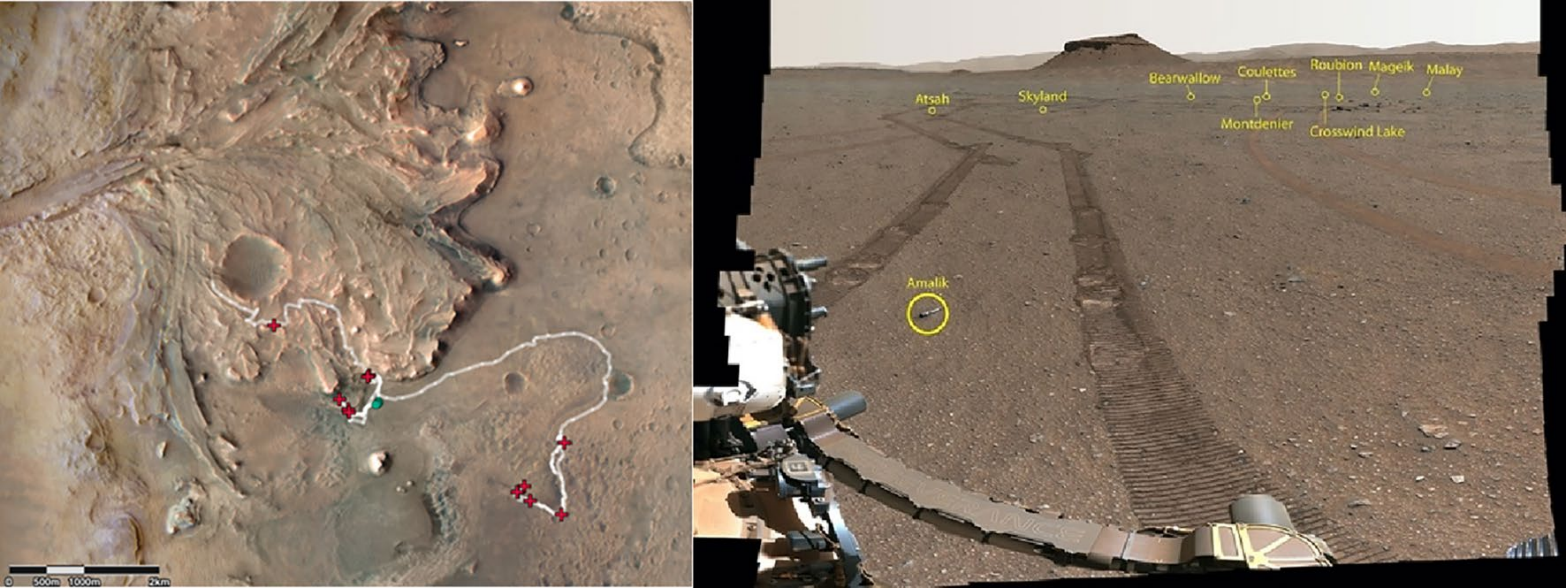
(Left) Perseverance’s traverse during the first 766 sols, from the landing site, through the Crater Floor and Delta Front campaign, and towards the western delta of Jezero crater, Mars. The white line indicates the rover traverse, green dots mark the deployment sites of the First Cache, and red crosses mark the sampling sites (including the sample sealed on sol 749, acquired above the delta after the construction of the sample depot). *Credit: CAMP and MRO HiRISE, The University of Arizona.* (Right) Annotated landscape of the Sample Depot at Three Forks, as seen by Perseverance, with the different sealed tubes. *Credits: NASA/JPL-Caltech/ASU/MSSS*.

The Mars Sample Return (MSR) program is a unique space project aimed at collecting a set of up to 38 samples and 5 witness tubes (or controls) from Mars with the *Perseverance* rover^1^, and is currently planning to retrieve up to 30 samples back to Earth by 2033^2,3^. Since February 2021, when *Perseverance* landed on Jezero crater [18.4663° N,77.4298° E] at solar longitude Ls = 5.2°, the rover has been exploring the surface of Mars and acquiring a collection of samples^4^. After the first Martian year of surface operation, 21 of these tubes were sealed as part of the “Crater Floor Campaign” (which ended on sol 380, where a “sol” is one rotation of Mars, i.e., a Martian day) and the “Delta Front Campaign” (which began on sol 415 and ended on sol 707, around mid-February 2023). Most samples were collected in pairs so that one sample from each pair was deposited on the ground forming the Sample Depot or First Cache at Three Forks^5^. The second sample in the pair was retained in the rover main collection. As the rover continues its exploration route towards the top of the delta fan and crater rim (Fig. 1), the sample cache increases in size and diversity with new added samples. The rover collection will be delivered in the future to the MSR sample receiving lander, while the Sample Depot at Three Forks would be used only if the rover failed before delivering its samples to the vehicle that will bring the samples to Earth. Upon reception on Earth of the sample collection, one of the first investigations to be implemented will relate to sample safety assessment and the search for Martian life in biocontainment^2,6,7^.

For planetary protection and life assessment purposes, there is a need to determine first the potential habitability of Jezero Crater's surface and the collection of samples that will be brought to Earth. Water is a requirement for known Earth life. On Earth, water activity, a_w_, is a measure of how much water (H_2_O) is free, unbound, and available for microorganisms to use for growth, and thus the habitability of an environment is restricted by the thermodynamic availability of water (i.e. the water activity, a_w_)^8,9^. The currently accepted lowest documented limit for life is a_w_ = 0.585^10^. This low level of water activity allows the germination of the xerophilic, osmophilic and halophilic fungus *Aspergillus penicillioides*. The present lower temperature limit for cell division is 255 K (− 18 °C) as reported by Collins and Buick^11^ in experiments with the psychrotrophic pink yeast *Rhodotorula glutinis.* For planetary protection purposes, some margin is added to this limit, and it is assumed that cell replication needs water activity a_w_ > 0.5 and temperatures T > 245 K (− 28 °C)^12,13^. These physical parameters are commonly used to assess at a planetary scale the habitability of a region and to define the planetary protection protocols and restrictions that should be applied to prevent forward contamination associated with space exploration missions^14,15^. To determine the potential present-day habitability of the surface of Jezero Crater, we will analyse these two environmental parameters: temperature and water activity and the possible interaction of atmospheric water (H_2_O) with salts. Similar analysis has been done previously at a planetary scale using global circulation models^16,17^ and at a local scale using in-situ environmental measurements at Gale Crater^18^ and Phoenix landing site^19,20^.

Salts were found at Jezero Crater in the abrasion patches associated with each sample^4^. Hygroscopic salts can absorb atmospheric water vapor (H_2_O molecules in gas state) to form liquid solutions (brines) in a process called deliquescence^21^. Additionally, salts in contact with the atmosphere can hydrate (solid-state hydration) and dehydrate, capturing and releasing H_2_O molecules. The plausible existence of brines or salt hydrates on the surface or subsurface has several implications for Mars's past and current habitability. Experiments in simulation chambers have shown that for certain temperature and a_w_ conditions, Mg, Ca, and Na perchlorates and sulfates can hydrate or deliquesce, forming stable liquid brines under present-day Martian conditions^22–24^. The Planetary Instrument for X-Ray Lithochemistry (PIXL) and the Scanning Habitable Environments with Raman and Luminescence for Organics and Chemicals (SHERLOC) instruments have investigated the abrasion patches and found hygroscopic and deliquescent salts such as Mg, Fe (hydrated) and Ca sulfates (anhydrite mostly), chlorides and perchlorates (Initial Reports-PDS;^25–28^). Also, the SuperCam (SCAM) instrument found that the visible/near infrared (VISIR) spectra of the abraded patches in the rocks of some of the sample pairs (the ones named Roubion, Montdenier, and Montagnac) are consistent with a mixture of hydrated Mg-sulfates, whereas SCAM Raman and Laser induced breakdown spectroscopy (LIBS) and SHERLOC detected anhydrous Na perchlorate^25,26,29^. Previous Mars exploration missions have detected Mg- and Ca-perchlorates at the Phoenix^30,31^ and Mars Science Laboratory^32^ landing sites. Amongst the salts found at Jezero, and on Mars, calcium perchlorate is the deliquescent salt that has the lowest eutectic point (198 K)^16,33^, and thus, this is the lowest temperature limit for liquid water (brine) stability of single component brines on present-day Mars. Sulfate signatures were detected in the SCAM VISIR spectra of the abraded patch of the sample named Bellegarde^26,29^ as well as in the Hogwallow Flats region explored in the Delta Front Campaign, which showed a hydrated sulfate-cemented siltstone^34^. Also, PIXL and SHERLOC detected sulfates in these environments. The presence of these different types of salts suggests that Jezero Crater was exposed to episodic water events, with different salt solutes that precipitated during evaporation^28,35–37^. Previous in-situ research by the Curiosity rover at Gale Crater has shown that sulfates are the main carrier of soil hydration^38^, which is consistent with orbital observations at the planetary scale^39^.

To characterize the near-surface water cycle at Jezero and the habitability of the Martian rocks that have been sampled, we need to quantify the amount of water that is available daily for exchange with outcrops and regolith, evaluate the potential hydration state of the salts that have been found on Mars and at Jezero and estimate the moles of H_2_O in the headspace gas of the sealed samples using the Mars Environmental Dynamics Analyzer (MEDA) instrument observations^40,41^, see Supporting Information A.

## Results

The collection of samples acquired during the first Martian year and the environmental conditions during the sealing are summarized in Table 1.

**Table 1:** Summary of acquired samples, sealing sol and Local Mean Solar Time (LMST), solar longitude (Ls), ambient temperature (Ta) at 0.84 m above the ground and pressure (P) provided by MEDA, estimated sample length (L), estimated rock volume (V), estimated rock mass (M) assuming a sample density of 2.6 g/cm^3^, estimated headspace gas volume (G), estimated total number of moles of gas (n) (micro-mol), Single Column Model (SCM)-derived H_2_O VMR at the time of sealing at 0.84 m above the ground, H_2_O partial pressure and derived number of moles (nano-mol) of H_2_O. The samples left on the ground at Three Forks as part of the First Sample Depot are shadowed in colour. WB# refers to witness tube assemblies, as described in ^59^.

M2020 sample	Sealing sol and time (LMST)	Ls	Ta (K)	P (Pa)	L (cm)	V (cc)	M (g)	G (cc)	Gas n (*µ*mol)	H_2_O VMR (ppm)	P H_2_O (Pa)	H_2_O n(*n* mol)
WB1	Sol 120, 16:25:55	62,07	245	758	0,00	0,00	0,00	6,00	2,23	60	0.045	0,13
Roubion	Sol 164, 19:11:35	81,87	221	749	0,00	0,00	0,00	12,00	4,89	30	0.022	0,14
Montdenier	Sol 194, 16:53:19	95,42	245	702	5,98	8,40	21,84	3,60	1,24	60	0.042	0,07
Montagnac	Sol 196, 20:35:07	96,40	215	707	6,14	8,70	22,62	3,30	1,31	20	0.014	0,03
Salette	Sol 262 20:22:00	127,54	221	643	6,29	8,87	23,06	3,13	1,10	50	0.032	0,06
Coulettes	Sol 271 20:18:00	131,98	222	636	3,35	4,72	12,28	7,28	2,51	50	0.032	0,13
Robine	Sol 298, 17:22:56	145,62	246	608	6,08	8,57	22,29	3,43	1,02	120	0.073	0,13
Malay	Sol 337, 19:25:37	166,59	223	642	3,07	4,33	11,26	7,67	2,66	50	0.032	0,14
Hahonih	Sol 371, 19:21:57	186,04	227	672	6,50	9,24	24,02	2,76	0,98	50	0.034	0,05
Atsah	Sol 377, 21:12:02	189,64	215	669	6,00	8,46	22,00	3,54	1,32	40	0.027	0,06
Swift Run	Sol 490, 21:23:31	261,36	208	817	6,69	9,43	24,53	2,57	1,21	23	0.019	0,028
Skyland	Sol 495, 21:22:05	264,61	210	808	5,85	8,25	21,45	3,75	1,74	40	0.032	0,069
WB2	Sol 499, 20:14:37	267,17	213	798	0,00	0,00	0,00	6,00	2,70	40	0.032	0,030
Hazeltop	Sol 509, 20:57:08	273,65	212	800	5,97	8,42	21,89	3,58	1,63	25	0.020	0,041
Bearwallow	Sol 516, 20:27:20	278,13	214	797	6,24	8,80	22,88	3,20	1,43	40	0.032	0,06
Shuyak	Sol 575, 19:39:58	314,59	218	750	5,55	7,83	20,35	4,17	1,73	50	0.038	0,09
WB3	Sol 586, 19:16:39	321,03	226	722	0,00	0,00	0,00	6,00	2,31	60	0.043	0.04
Mageik	Sol 619, 19:53:14	339,68	225	725	7,36	10,38	26,99	1,62	0,63	60	0.044	0,04
Kukaklek	Sol 631, 18:16:17	346,15	237	701	4,97	7,01	18,22	4,99	1,78	90	0.063	0,16
Atmo Mountain	Sol 634, 22:16:11	347,85	210	722	5,30	7,47	19,43	4,53	1,87	30	0.022	0,056
Crosswind Lake	Sol 639, 21:30:01	350,49	211	727	5,30	7,47	19,43	4,53	1,88	50	0.036	0,09

**Figure 2:**
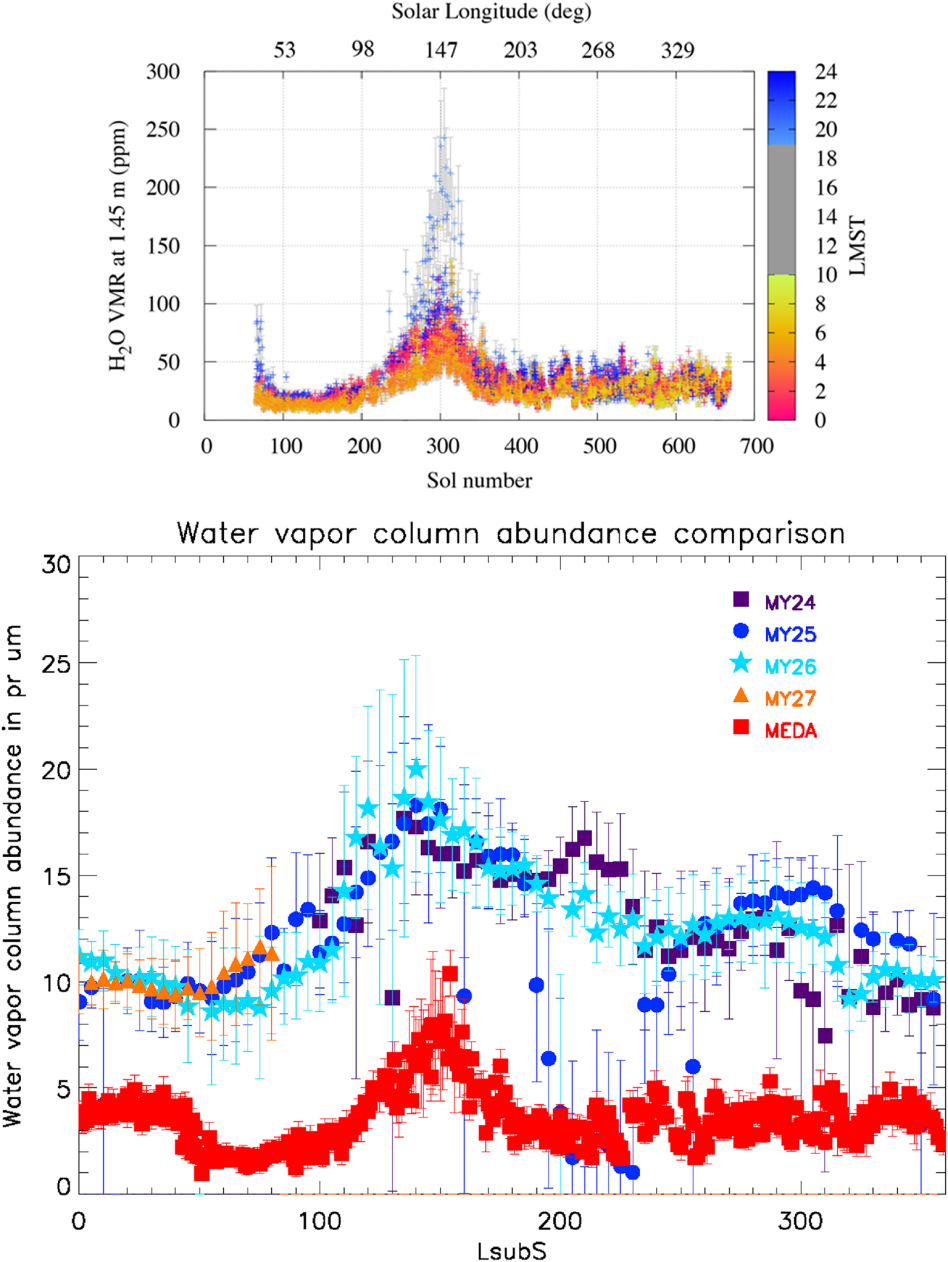
(Top) Annual (sol number and Ls) and night-time (LMST) variation of the Water Volume Mixing Ratio (VMR), with error bars, at Jezero crater during the first Martian year provided by the MEDA instrument at 1.45 m above the surface. Daytime relative humidity measurements (marked in gray) fall below the 2% accuracy of the MEDA relative humidity sensor and the VMR cannot be estimated. The spring equinox starts at L_s_ = 0°, the summer solstice at L_s_ = 90°, the autumnal equinox at L_s_ = 180°, and the winter solstice at L_s_ = 270°. (Bottom) Total column of H_2_O abundance (in precipitable microns): TES zonally-averaged orbiter data for MY24 to MY27 (daytime, ~ 14 LMST) compared with MEDA (pre-dawn) in-situ surface measurements (lower data set) at Jezero Crater. For orbital data, the error bars are the 1-sigma standard deviation on the average that is plotted. MEDA error bars are derived from the MEDA reported uncertainty value in the relative humidity (RH) measurements and in the humidity sensor board temperature.

The annual and diurnal variation of the water vapor volume mixing ratio (VMR) at Jezero crater is shown in Fig. 2 using MEDA observations^42^. Daytime MEDA Relative Humidity (RH) measurements are too low (i.e., ≤ 2%, the RH uncertainty) and thus cannot be used to estimate VMR with sufficient accuracy. MEDA relative humidity and pressure measurements at 1.45 m above the surface suggest a strong diurnal and seasonal variability of the water VMR, see Fig. 2-Top. The water volume mixing ratio peaks at Ls = 150°, at the end of the northern hemisphere summer after the release of water vapor from the northern polar cap. Predawn MEDA measurements (when the confidence in VMR retrieval is higher) have been used to estimate the (total column) night-time precipitable amount of water. The results are compared with the daytime zonally averaged orbital observations provided by the Thermal Emission Spectrometer (TES) onboard the Mars Global Surveyor orbiter for this region in Fig. 2-Bottom. There is coherence in the seasonal behavior, the zonally averaged orbital daytime observations and the in-situ nighttime observation differ by a factor of 2–3. According to MEDA in-situ night-time measurements, the greatest amount of nighttime precipitable water is around 10 pr-um at Jezero crater, and was reached around Ls = 150°, during the northern hemisphere summer, around the sampling time of Robine. A precipitable micrometer (pr-μm, which equals 1 g of H_2_O per m^2^) is the thickness of the water layer that would be condensed on the surface if all the water vapor of the corresponding atmospheric column would accumulate on the surface. Orbital and in-situ measurements have been compared with a Global Circulation Model, see Supporting Information D, and the annual trends are in agreement.

**Figure 3:**
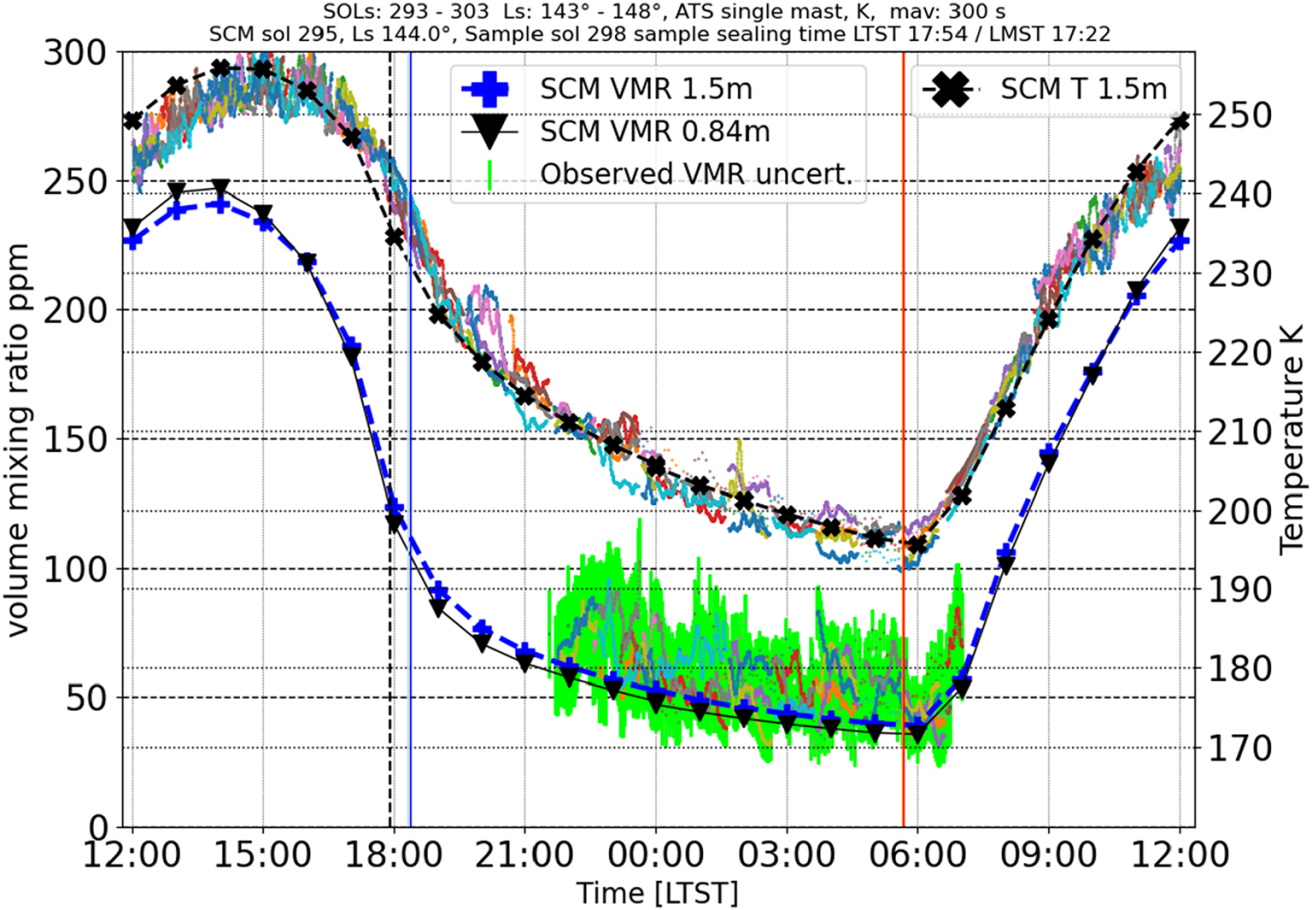
Near-surface diurnal cycle of water Volume Mixing Ratio (VMR) and air temperature (T) as a function of LMST during the sols around the sampling time of Robine. Single-column model (SCM) VMR results—dark and light blue lines—at 1.45 m and 0.84 m, respectively, are compared to MEDA values (including the uncertainty in H_2_O VMR retrieval) at 1.45 m for sols 285 to 305 (Ls = 139°–149°). The SCM air temperature estimate—black line—for the same period compared with the Air Temperature Sensor (ATS) observations at 0.84 m (with 300 s moving average). The time of sealing is marked with a vertical dashed black line, whereas sunset and sunrise times are marked with a blue and orange line, respectively.

An example of the amplitude of the diurnal variability of the near-surface H_2_O content is illustrated in detail in Fig. 3. Here we compare the nighttime H_2_O VMR values of several consecutive sols (sols 293 to 303, around the sampling sol of Robine at Ls = 146° at the end of the northern hemisphere summer) with the results of the Single Column Model (SCM). The SCM provides an estimate of the diurnal H_2_O VMR and can also be used to extrapolate the VMR value at the height of the sealing station (around 0.84 m, where two other MEDA temperature sensors are). The corresponding air temperature measurements at 1.45 m, through day and night, are also included for completeness. This example shows, for instance, a diurnal variability of H_2_O VMR of a factor of 5 or more; in this case, the H_2_O VMR ranges between 40 and 240 ppm. The lowest ground temperatures are reached just before sunrise; at this moment, the relative humidity of the ground peaks, and sometimes frost conditions can be met when saturation is reached. This is confirmed by measurements and models (see Supporting Information B).

**Figure 4:**
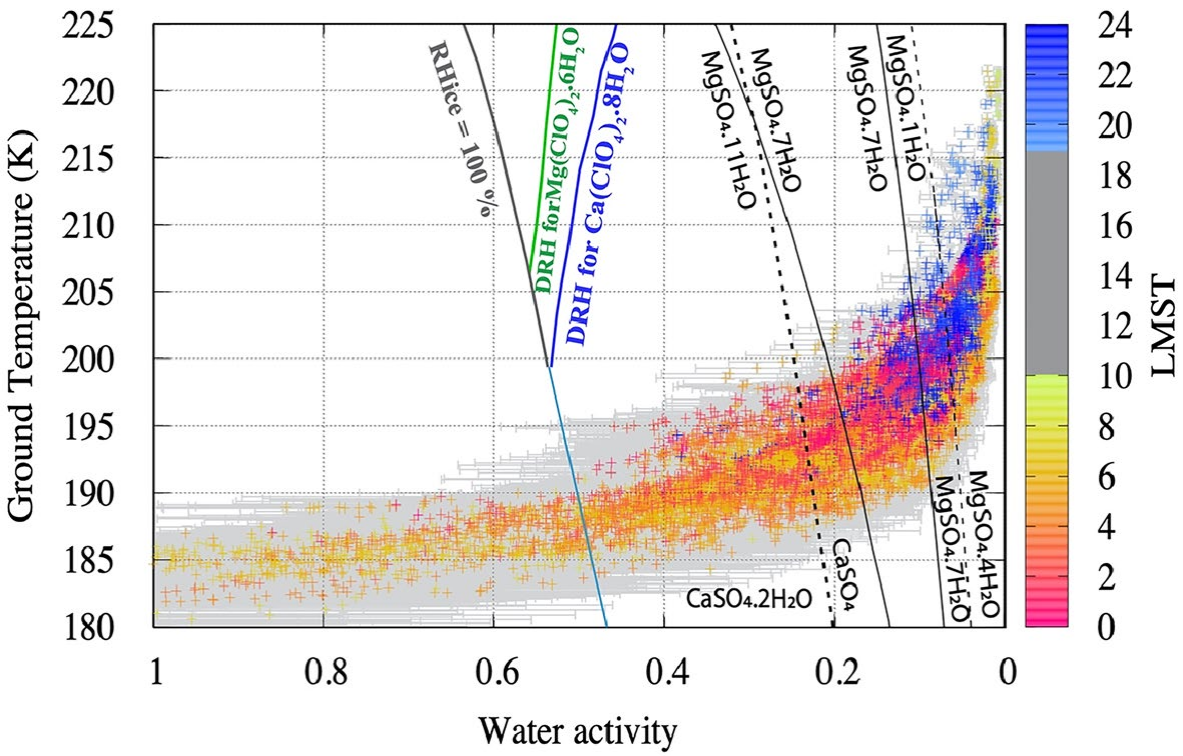
Diurnal variation, as a function of LMST, of the derived surface water activity concerning liquid (with a_w_ error bars) and measured ground temperature provided by MEDA during one full Martian year. For illustration, the environmental data are overlayed with the hydration lines of calcium and magnesium sulfates, and calcium perchlorate deliquescence and efflorescence lines. The water activity a_w_ is derived assuming equilibrium, from the relative humidity (RH), with respect to liquid, as a_w_ = RH/100, All data points to the left of the ice saturation line (RH_ice_ = 100%) are saturated with respect to ice and may allow frost formation^70^. The Deliquescence RH (DRH) and hydration state lines of some perchlorates and sulfate salts are included for reference^19,72^.

On the surface of Mars, there is a strong anti-correlation between water activity and temperature, as illustrated in Fig. 4. All other factors being equal, for the same amount of water VMR, the relative humidity increases with decreasing temperature. Although MEDA surface measurements suggest a factor 5 reduction of the water VMR at night-time, the large temperature decrement overcomes this and results in an increased night-time relative humidity (and water activity). Figure 4 shows the pairs of (simultaneous) derived groundwater activity and measured ground temperature (with accuracy 0.75 K) as measured by MEDA instrument throughout the night during one full Martian year at the base of Jezero crater. This analysis is shown in the Supporting Information E, divided into four seasons. The values are compared with the known phase and hydration state changes of some of the salts reported in the abraded patches. The deliquescence curve for calcium perchlorate (the salt found on Mars with the lowest eutectic temperature, 198 K) is also included for reference.

**Figure 5:**
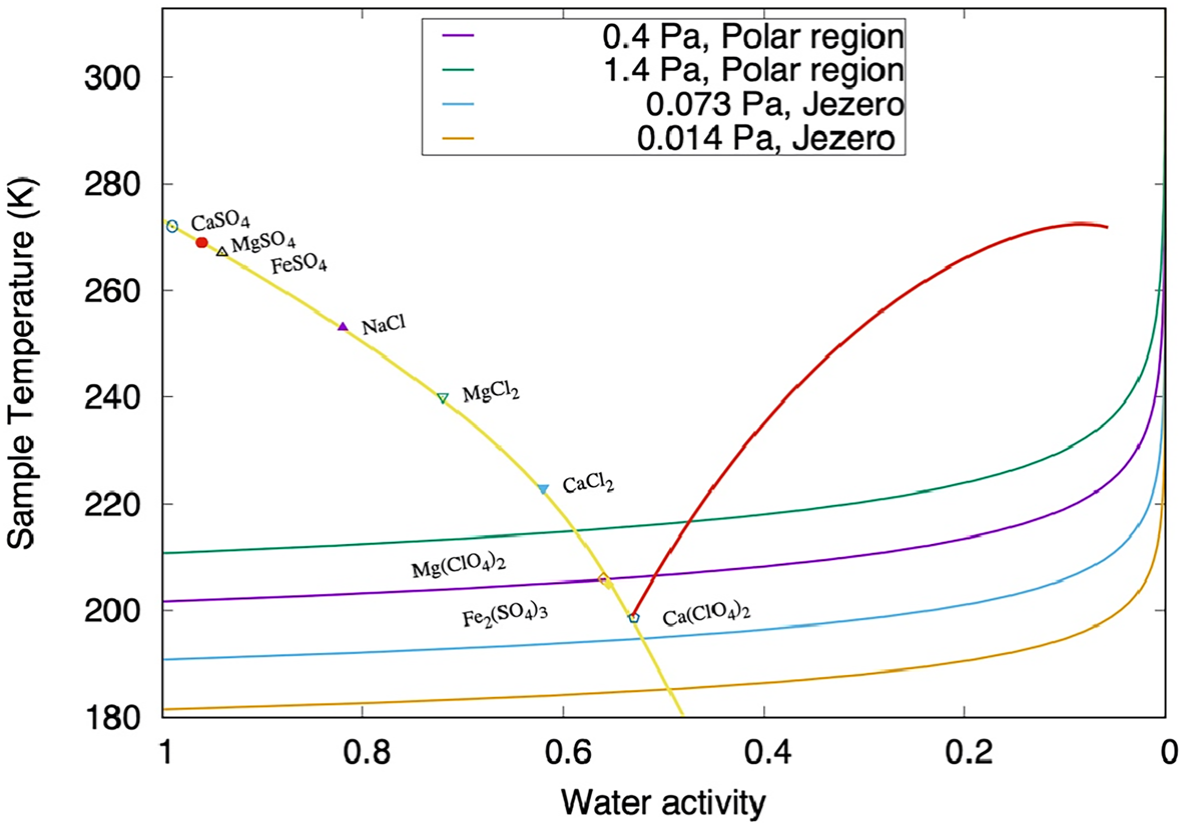
Modelled thermal-water activity curves experienced by the samples within the sealed tubes. The H_2_O partial pressure isobars (i.e., constant water vapor pressure) for the higher and lower partial pressure reported in Table 1 are compared with the eutectic points of different salts of relevance to Mars, which may be within the sampled rocks (colored symbols), the temperature-dependent deliquescence relative humidity (DRH) for calcium perchlorate (red line), and the ice liquidus line (i.e., equilibrium between water ice and liquid brine; light yellow)^17,70,73^. For comparison, the isobar for the H_2_O partial pressure values that are expected at polar regions, i.e. 0.4 Pa and 1.4 Pa^19^, is also included.

Once the samples are sealed, they may experience changes in water activity caused by exposure to different thermal environments (either on the surface of Mars, within the rover, during the launch, cruise, entry, descent and landing phases, or during storage on Earth). For illustration we have modelled a simplified, T/ a_w_ cycle for the gas space of a sealed sample (Fig. 5) assuming a range of possible temperature changes experienced by the samples on Mars, on the rover or on its way to Earth. We assume that the water VMR is constant in the tube and equal to that in the atmosphere when the samples were sealed. We take this assumption because the type and amount of salts captured within the bulk of the 3–6 cm deep drilled core is not exactly known. Therefore, it is not possible to accurately simulate how much captured water will be released from the core salts into the headspace gas when the sample tubes are heated. We compare the isobaric lines, for the higher and lower partial pressure reported in Table 1, with the eutectic points of different salts of relevance to Mars, which may be within the sampled rocks. All isobars pass below the eutectic points of these salts, suggesting that if there are no additional water sources in the rock samples, no pure salt would deliquesce (although mixtures of salts may behave differently).

## Discussion

Within the first Martian year, Perseverance has acquired an estimated total mass of 355 g of rocks and regolith, and 38 μmole of Martian atmospheric gas (Table 1). A preliminary MSR study estimated that the atmospheric sample needed to implement volatile studies should be at least 19 μmole^43^, ideally within one single dedicated tube. The First Sample Cache, which constitutes a contingency collection formed by a set of 10 sample tubes, contains a total of 21 μmole of gas and 158 g of rock mass. The amount of gas available at the First Sample Depot meets the requirement of gas amount proposed by Swindle et al.^43^, although the gas is distributed within the headspace of different sample tubes, the witness tubes and in one dedicated “atmospheric” sample (Roubion). The water content in the sealed gas varies from sample to sample, depending on the sealing time and season.

The analysis of atmospheric data from one full Martian year suggests that the surface at Jezero crater can act as a water sink at night, with most of this water released back into the atmosphere after sunrise. The combined analysis of orbital and in-situ measurements suggests that there is a strong diurnal cycle whereby the near-surface water VMR changes by a factor of 3–5, which agrees with previous observations by Curiosity at Gale Crater, Mars^44^. Comparing day-time orbital and night-time surface observations, and assuming that the entire atmosphere participates in the interchange, we conclude that the maximum amount of water potentially available for this daily interchange is around 10 pr-µm, although a value near 0.5 pr-µm is more likely since models indicate that only the lowest ~ 200 m of the atmosphere directly exchanges with the surface on a diurnal timescale^45,46^, see Supporting Information D. Notice that this assumes a well-mixed atmosphere up to a certain height. This means that the diurnal cycle of water may thus allow for a daily transfer of about 0.5 g of water per m^2^ (assuming H_2_O is well-mixed within the lower 200 m) with an upper limit of as much as 10 g m^−2^ (assuming H_2_O is well-mixed up to the scale height). Previous analysis of the vertical profile at arctic Martian regions suggests that during spring and summer, a large percentage of the water column (> 25% and up to nearly 100%) was confined below ~ 2.5 km^47^. These results are comparable to those provided by the REMS instrument package on the Curiosity rover at Gale crater^24^ and are consistent with previous research based on orbital and in-situ observations and modelling^44,48–54^. We conclude that similarly to what happens on other sites on Mars^55^, there is a strong rock and regolith-atmosphere exchange mechanism on Mars^56^, likely owing to the combination of adsorption–desorption of water on the regolith grain surfaces and to hydration-dehydration of salts.

The present-day surface water activity and temperature cycle at the surface in Jezero does not allow the formation of deliquescent brines (although it may happen in the subsurface, should kinetics allow). During some periods of the year, the surface relative humidity is saturated with respect to ice, and frost can be transiently stable for some hours of the day when the ground temperature is below 185 K. The present-day surface environment at Jezero allows hydration and dehydration of different forms of salts on a diurnal and seasonal basis, as illustrated in Fig. 4. Our analysis suggests that the daytime environmental conditions allow for MgSO_4_.4H_2_O stability. Indeed, the analysis of PIXL and SHERLOC data of the abraded patches has found hydration (3–5 waters) in association with the Mg sulfate salts^27^, which is in line with the analysis of Fig. 4. The regolith at Jezero crater has been investigated by the Planetary Instrument for X-ray Lithochemistry (PIXL) and SuperCam LIBS and VISIR instruments^56^. Their analysis has demonstrated that the top surface of soils, which is the part in direct contact with the atmosphere, is enriched in water and S and Cl salts that form a crust. Some targets showed a strong correlation between S, Mg, and H, suggesting the presence of Mg sulfates, which are likely hydrated. Note that the crust hydration signature is seen even during daytime when the ambient relative humidity and water activity are below 0.02, which indicates that water is not released immediately to the atmosphere due to the slow kinetics of dehydration.

The sustained hydration/dehydration cycle of salts at Jezero, within the rock matrix, exposed to this environment for millions of years may have induced the formation of voids and cracks in the rocks and may have contributed to their mechanical erosion and disaggregation^35^. Salt hydration and dehydration can indeed cause substantial volume expansion; for example, magnesium sulfate can increase its volume by up to 70%^57^, generating substantial stresses and weakening the rock^58^. Interestingly, the first abraded patch (Roubion sample), showed voids of millimetre to centimetre size, which were not visible on the rock surface. The composition analysis of Roubion abraded patch revealed that Ca- and Mg-sulfates, Ca-phosphates, and halite were present in significant concentration. In this rock, Na-perchlorates constituted more than 60% percentage out of the total SHERLOC mineral detections^25^. The sample from Roubion rock completely disintegrated during drilling, suggesting that due to this environmental cycle salt-rich samples may be fragile and disaggregate during their future mechanical manipulation on Earth.

Documenting the water content is important for sample integrity to estimate what may happen to the samples on their way to and during manipulation on Earth. When the samples are sealed, they will equilibrate over time with their headspace gas. The hydration state of the samples within its sealed capsule depends on the temperature during storage in the rover, or on the surface, or during cruise, or entry or final storage on Earth. Most of these temperatures will have to be measured, inferred, or modelled. For instance, once on the surface of Mars, the tubes may potentially, repeatedly, be heated ocationally to up to 300 K for years. Also, their minimum night-time temperatures will presumably be similar to the surrounding regolith (about 180 K), see Supporting Information C. The sample tubes are coated in alumina (white) and titanium nitride (golden parts)^59^. These coatings can interact with the incident solar radiation during the day absorbing radiation, and at night with the atmosphere above emitting infrared radiation, resulting in local temperatures that may differ slightly from the one of the natural bedrocks and regolith Martian surface, see Supporting Information C. As for the samples within the rover they will be exposed to a different thermal history. For illustration we have modelled a simplified, T/ a_w_ cycle for the gas space of a sealed sample (Fig. 5). At first order, assuming equilibrium and a well-mixed atmosphere, all the isobars pass underneath the eutectic points of single salts relevant to Mars.

Based on the currently recognized limits of known life forms on Earth, cell replication requires temperatures above 245 K (− 28 °C), and -simultaneously-water activity above 0.5^12^. During all seasons, the water activity at the ground surface at Jezero crater can frequently go above the limit for terrestrial cell reproduction of 0.5, but this happens only at night, when the temperature at the surface drops below 190 K (Fig. 5). Therefore, the present-day Mars surface conditions at Jezero crater are very different from the known, tolerated limits for cell replication on Earth. The limits used as reference for Planetary Protection Policies are documented in laboratory growth studies that confirmed cell reproduction. There are extremely arid subsurface natural environments on Earth, e.g., the Atacama Desert’s Maria Elena South region, where, at a depth of a few dm’s, the water activity is constantly of the order of 0.14 (i.e., 14% RH). It has been shown that in this subsurface hyper arid environment, there still is as much microbial diversity as at the surface where the mean water activity value is 0.27^60^. However, in this region but the temperature never reaches 245 K. The environmental conditions at Jezero crater are inadequate for deliquescence but allow for hydration of Ca and Mg sulfates, among other salts. On Earth, some recent studies used gypsum (CaSO_4_·2H_2_O) samples collected in the Atacama Desert as a substrate for culture experiments with a cyanobacteria strain. This research demonstrated that cyanobacteria could extract water of hydrated salts from the rock, inducing a phase transformation from gypsum to anhydrite (CaSO_4_), which may enable these microorganisms to sustain life in this extremely arid environment^61^. The validity of these results has been questioned^62^, which suggests that the existence of water extraction mechanisms from salts and dry rocks across other organisms needs to be further investigated to understand better the limits of life on Earth and Mars^63^.

Based on the state-of-the-art research of the limits of life tolerance on Earth, we conclude that the samples' environmental conditions at Jezero Crater are incompatible with the known cell replication requirements. If future research of life on Earth demonstrates low-temperature cell replication using the water of hydrated sulfates or water adsorbed to rock grains, then the habitability of the Martian sample collection should be reassessed, as day-time temperatures at Jezero are compatible with cell replication.

## Methods

Once a sampling target was identified during the rover’s surface operations, a 5 cm diameter patch was abraded within a few tens of cm of the desired sample targets, within the same lithology, to remove surface dust and coatings. In this abraded patch, which was taken as proxy for the sample, detailed images of rock textures and maps of elemental composition, mineralogy and organic molecule distribution were acquired with the rover instruments. Samples were acquired with drills and were afterwards sealed at the rover sealing station. Prior to sealing, the length of the solid cores is estimated by Perseverance using a volume probe^59^. Each tube has an internal volume of 12 cm^3^ (with a tube section of 1.4103 cm^2^). Witness tubes are assumed to have only half of their internal volume available for gas. The Initial Reports have documented all the details of sampling acquisition and instrument observation interpretation^35^ (2023).

Table 1 indicates the sealing sol (starting on the first day of Perseverance on Mars operations) for each sample. The measured sample length and MEDA atmospheric temperature at 0.84 m above the surface (Ta) (which is comparable to the height of the sealing station) and atmospheric pressure (Pa) (see supporting information A), are used to calculate the total mass of rock (M), assuming a sample density of 2.6 g/cm^3^ (the same one used in the Initial Reports-PDS), and the estimated partial pressure of water and number of moles of gas (n) in the headspace above the solid sample. Local Mean Standard Time (LMST) indicates the time when the sealing was activated. The solar Longitude (Ls) marks the passage of time within a Mars year and the changes through seasons.

For consistency, in the mass calculation of Table 1 we have applied to all samples the same density used in the Sample Reports (2.6 g/cm^3^). But the actual density of each sample may vary significantly. For instance, the bulk density of regolith granular material on Mars has been estimated to range between ∼1 and 1.8 g/cm^3^^64^,the density of the bedrock at Jezero through the traverse of the rover has been estimated, based on RIMFAX radar measurements, to vary between 3 and 3.4 g/cm^3^^65^ whereas using SuperCam mineral abundances, the densities of some of the targeted rocks on the crater floor have been inferred to vary between 3.1 and 3.7 g/cm^3^^66^. As for other rock types, the density of sedimentary rocks in Gale crater have been calculated to be of the order of 2.3 ± 0.130 g/cm^3^^67^. We use a single-density value of 2.6 g/cm^3^ for all samples, which is an average of the densities of these three rock types (dense bedrock 3.7 g/cm^3^, sedimentary 2.3 g/cm^3^ and regolith 1.8 g/cm^3^).

The environmental information at the time of sealing is recorded by the Mars Environmental Dynamics Analyzer (MEDA) instrument package (MEDA Data;^40^). During the sample sealing process, each tube was heated up to 40 °C (313 K) for a short period of time (minutes) as recorded by the PRT temperature sensors at the time of sealing. This does not translate to heating the sample itself to such temperature, but it is considered an upper temperature limit that the samples should not exceed. The actual temperature inside the sample tube during sealing is likely between MEDA ambient temperatures and the Platinum Resistance Thermometer (PRT) measurements. MEDA also measured the ambient pressure and temperatures (for more information on the measurement cadence, see Supporting Information A). The sample length probe is used to estimate the rock volume, and the remaining headspace volume is occupied by Martian atmosphere gas, then the temperature and pressure provided by MEDA, are used to calculate the number of moles of the sealed headspace gas. All this information is included in two main products that are uploaded to the NASA Planetary Data System (PDS): (1) the Sample Dossier, that contains all observations from the instrument payloads at the sampling site, along with relevant rover ancillary data; (2) and the Initial Report, which is an extended description of the observations of each sample prepared by the Science Team within a few weeks of sample acquisition (K.A. Farley and K.M. Stack, Mars 2020 Initial Reports—Crater Floor Campaign, 2022; K.A. Farley and K.M. Stack, Mars 2020 Initial Reports—Delta Front Campaign, 2023).

Water activity is defined as the equilibrium fugacity of water vapor over a solution (f) relative to the fugacity of water vapor over pure water (f_0_) (a_w_ = f/f_0_). At low pressures, such as on Mars, fugacities are well approximated by partial vapor pressures, leading to the more common expression a_w_ = e/ e_s,w_ (T_g_), where e_s,w_ is the saturation vapor pressure over liquid water, which is equivalent to the equilibrium relative humidity (RH) divided by 100 (RH/100 = a_w_)^9^. We use MEDA’s Relative Humidity Sensor (HS) and Thermal Infrared Sensor (TIRS) to derive the water activity at the ground and to measure ground temperature^42,68^. The HS measures the relative humidity (RH) with respect to ice at 1.45 m with an uncertainty of 2%. For a detailed explanation of the RH, the retrieval procedures and error sources see^69^, and the measurements acquired during the first 410 sols of operations^42^. The HS can also be used to estimate the water vapor pressure at 1.5 m as e = RH × e_s,i_(T_b_), where e_s,I_ is the saturation vapor pressure over ice that can be calculated theoretically for the measured T_b_, the temperature of the RH sensor board from the HUMICAP ® chip. Similarly, the water vapor volume mixing ratio at 1.45 m can be estimated as VMR = e/P, where P is the atmospheric surface pressure measured by MEDA. The HS output is only reliable above 2% and thus can only be used to retrieve water contents at local times ranging from ~ 20:00 to 07:00, with some seasonal variation^42^. For a detailed explanation of the RH, the retrieval procedures, error sources, and the measurements acquired during the first 410 sols of operations see^42^. The TIRS is located on the rover sensing mast at 1.5 m above the ground, with an orientation of 75° clockwise in the horizontal plane with respect to Z-axis local frame (with + X defined along the forward direction and + Y pointing to the right of the rover). TIRS measures the surface brightness temperature (T_g_) in the 8–14 µm range with a downward looking channel covering an ellipsoid area of 3–4 m^2^, and with an accuracy of 0.75 K and a resolution of 0.08 K^68^. Using TIRS and HS measurements, we calculate the water activity at the, i.e. a_w_ = e / e_s,w_ (T_g_), where e_s,w_ is the saturation vapor pressure over liquid water, that is also calculated theoretically in this case as a function of the measured ground temperature T_g_. We note that to calculate a_w_, we have assumed that the water vapor pressure is uniform in the first 1.5 m. Since the ground acts as a water sink, and vapor is adsorbed onto the ground at night, water vapor pressure at 1.5 m may be larger than at the ground. Therefore, for each instant of time, the reported a_w_ represents an upper bound of the actual water activity at the ground. We compare the temperature T and water activity a_w_ with the phase state diagram of some salts relevant to Jezero and Mars^17,70–73^. A note of caution is needed: as explained above, under equilibrium conditions (e.g., when a brine or a hydrated salt has equilibrated with ambient air and is not evaporating), water activity is equivalent to the relative humidity with respect to liquid; however, it is debatable if equilibrium can be reached between the atmosphere, the regolith and rock, and the salts, under the rapidly varying Martian surface conditions. Some research suggests that brine formation on Mars may be hindered by kinetics^48,74^, whereas other experimental work has confirmed that hydration and deliquescence can take place within a few hours under Martian representative pressures and temperatures^20,22–24^.

The column abundances shown in Fig. 2 (Bottom) for Mars Years 24–27 are from the Thermal Emission Spectrometer (TES;^75^) that flew on the Mars Global Surveyor spacecraft (MGS). MGS was in a near-polar orbit with a daytime LMST of ~ 14:00. The TES derived water column abundance (CA) values from the (non-normalized to constant surface pressure) climatology dataset is given in 3° latitude, 7.5° longitude, and L_s_ = 5° averaged bins for each Mars Year for which the TES experiment collected data. Thus, the latitudinal bin encompassing Jezero crater is 18°–21° N latitude. The CA were zonally averaged. The maximum MEDA RH values for each L_s_ were selected and converted to the mass mixing ratio (MMR). The equivalent CA were calculated, assuming a constant MMR throughout the atmosphere, using W = [q * ΔP]/g, where W is the mass of water in a column of unit area 1 m^2^, q is the MMR, g is Mars gravity, and ΔP is the atmospheric pressure. Then, CA = W/ρ, where ρ is the density of water in its condensed phase. For non-normalized mass mixing ratios, the ratio of CA to MMR is 4.9. This provides an upper limit on the diurnal variation of column water vapor since model results indicate that only the lowest ~ 200 m of the atmosphere participates in the strong diurnal variation in water vapor VMR observed by MEDA^45,46^. This may also explain the discrepancy between the TES and MEDA water vapor columns shown in Fig. 2 (Bottom) since the actual water vapor VMR is likely much greater above 200 m altitude than the low pre-dawn values measured by MEDA.

A single-column model (SCM) was used to extrapolate the night-time measured VMR values to daytime near surface VMR and to calculate the estimated amount of water in the headspace gas at the time of sealing. The adsorptive single-column (SCM) model has been described in detail before and applied to other in-situ observations on Mars^51–54^. Derived data and SCM model data are available in the Finnish Meteorological Institute repository. The results are successfully compared with the night-time MEDA measurements. The SCM-daytime VMR model has been used to calculate the headspace gas’s water content (VMR) during the sealing time (between 16:25 and 22:16 in LMST, see Table 1). There were four samples in which MEDA-HS measurements were available at the time of sealing, so the observed VMR value was used with an uncertainty of about 9 ppm. For the other samples the VMR is calculated using the SCM model, which fits the measurements of nearby sols, as in Fig. 3. The SCM gives an estimate valid as an average (lacks single sol and time precision). Thus, these values are rounded to the first significant figure. Knowing the remaining head-space volume and the water VMR, the number of moles of water can be calculated for each sample.

The annual and daily variations of the water content as measured by MEDA have been compared with predictions from the Mars Planetary Climate Model (PCM), developed at the Laboratoire de Météorologie Dynamique (LMD,^45^), by using observations over the Martian Year 36 (including the dust scenario of MY36) to reconstruct the simulated spatial and vertical dust distributions (thus representative of the conditions encountered by Perseverance during its first Martian year). At solar longitude Ls = 144° and at the location of Jezero (a box of 5° × 5° in the GCM), we extracted the water vapor volume mixing ratio at 5 m above the surface (this is a limit of the model and may induce some differences concerning the MEDA-measurements at 1.5 m), and the column mass of water vapor (in pr-µm). The climatology of airborne dust for year 36 was obtained using observations of the Martian atmosphere by the Thermal Emission Imaging System (THEMIS) aboard Mars Odyssey, and the Mars Climate Sounder (MCS) aboard Mars Reconnaissance Orbiter (MRO) ^76^.

### Supplementary Information

Supplementary Information.

## Data Availability

Initial Reports-PDS: Archive at the Planetary Data System, Geoscience Node, for samples 1 through 10, and samples 11 through 21: https://pds-geosciences.wustl.edu/missions/mars2020/returned_sample_science.htm. K.A. Farley and K.M. Stack, Mars 2020 Initial Reports Volume 1 Crater Floor Campaign, 2022. InitialReports Vol.1 and. K.A. Farley and K.M. Stack, Mars 2020 Initial Reports—Volume 2 Delta Front Campaign, 2023. InitialReports Vol.2. MEDA data: Archive at the Planetary Data System, Geoscience Node https://pds-atmospheres.nmsu.edu/PDS/data/PDS4/Mars2020/mars2020_meda/. Polkko, J. and Savijärvi, H. Finnish Meteorological Institute repository SCM Data: 10.57707/fmi-b2share.49d9d75e19c842b6b3e546ff1ae93649.
