# Supplementary material for: Present-day thermal and water activity environment of the Mars Sample Return collection
Source: Sci Rep. 2024 Mar 26;14:7175. doi: 10.1038/s41598-024-57458-4 (PMC10965995; doi:10.1038/s41598-024-57458-4)
Supplement: Supplementary file 1 — Supplementary Information. [file 41598_2024_57458_MOESM1_ESM.docx]

**Supporting information, A**

Sample tubes were sealed in the Adaptive Caching Assembly (ACA). Mechanisms in this subsystem are actively heated up to a fixed upper limit, so sealing occurred at temperatures higher than ambient conditions around the rover. The temperature recorded in the initial reports as ACA temperature was 313 K, which is the average of the temperatures recorded on the Sealing Station and the Sample Handling Arm end effector. These temperatures are likely warmer than the sample and the associated head space gas. The initial report and this work take as nominal reference the estimated temperature of the atmosphere in the vicinity of the rover as provided by the MEDA Air Temperature Sensors 4 and 5, which are located at an equivalent height above the ground surface. The reported temperature is the minimum of MEDA ATS 4 and 5, the sensors located 0.84 m above the surface. Atmospheric pressure is also obtained from MEDA.

When MEDA data were not acquired during the sealing time on the same sol as sample sealing (often the case given energy limitations), the average values of pressure and temperature are estimated from interpolation of the air temperatures measured around that period during the closest sols. These data gaps were especially big during the sealing at sols 164, 271, 495, 516, 586, 619 and 631. Figure S1 shows an example of the environmental evolution after 19:00 LMST, as acquired by MEDA measurements, during the sol of sample acquisition, and the closest sols before and after. Hahonih was sealed on sol 371, 22 minutes after 19:00 LMST, and Atsah on sol 377, 132, minutes after 19:00.

The estimated amount of headspace gas in moles is computed from the ideal gas law and assuming the rover ambient temperature (T) and pressure (P) described above.

n= P (V_tube_ - V_rock_)/ RT where R is the gas constant.

The total volume of the tube is assumed to be V_tube_ =12 cm^3^.

The actual temperature of the gas upon sealing is difficult to estimate because the ACA station is substantially warmer than rover surroundings, typically by almost 100 K. A lower gas density (and number of moles) limit would be obtained by using instead the reported ACA temperature in the above equation (313 K).


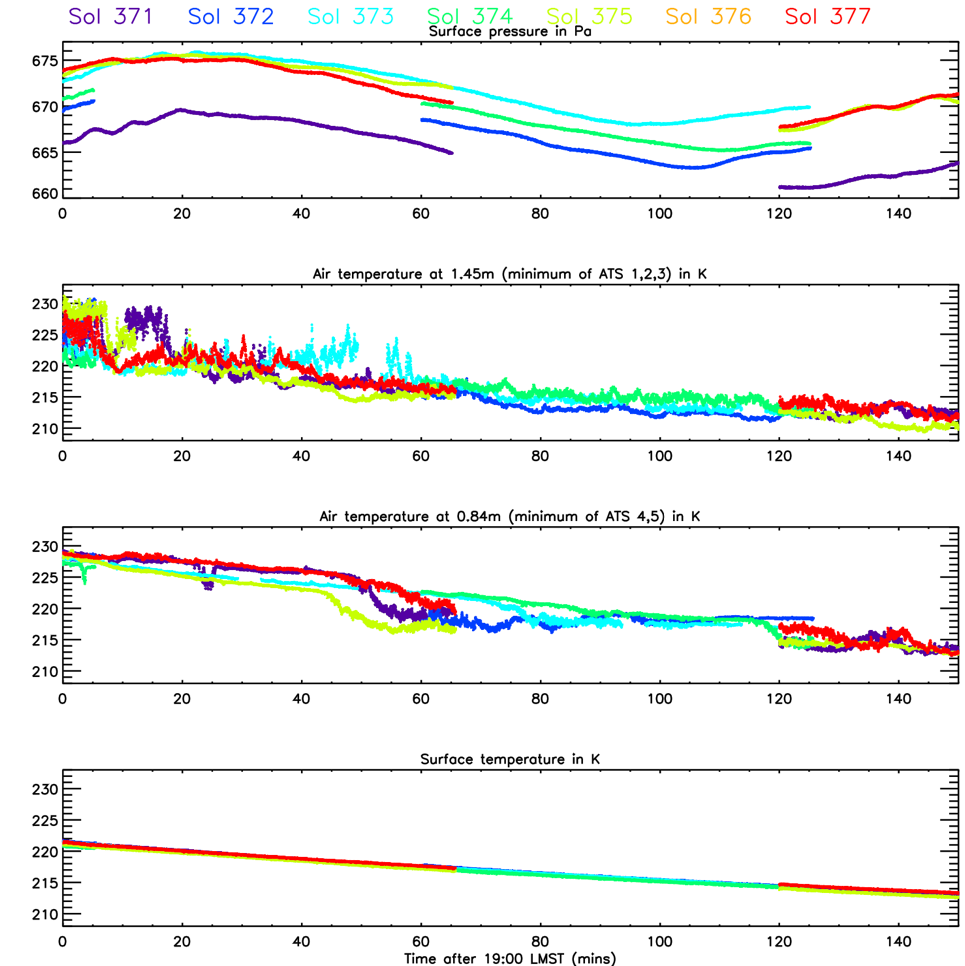


**Figure S1**: **Example of accumulated MEDA data** for the window of time around the sealing of Hahonih & Atsah (sol 371 at 22 mins and sol 377 at 132 min). The temperatures that are used for the headspace gas calculations are P and ATS_4-5.

**Supporting information B**

Figure S2 shows water activity (a_w_) with respect to ice from sols 250 to 530. Most of the sudden changes in night-time Aw are associated with drives of the rover, exploring different areas with different thermal inertias and diurnal temperature ranges. However, there are two periods when Aw tends to increase: the first one is from L_s_=100° to roughly L_s_=150°, when there is an increase in the atmospheric H_2_O VMR due to the global circulation of atmospheric water. The second period is around L_s_=270°, when the ground temperatures decrease with the arrival of winter and as result the relative humidity and water activity increase. During those periods, different sets of SCAM VISIR scans were acquired at the Dourbes, Quartier and Thornton Gap abraded patches, over the span of 19, 9 and 12 sols respectively. See the Initial Reports, for more information about the VISIR spectra of the abraded patches.


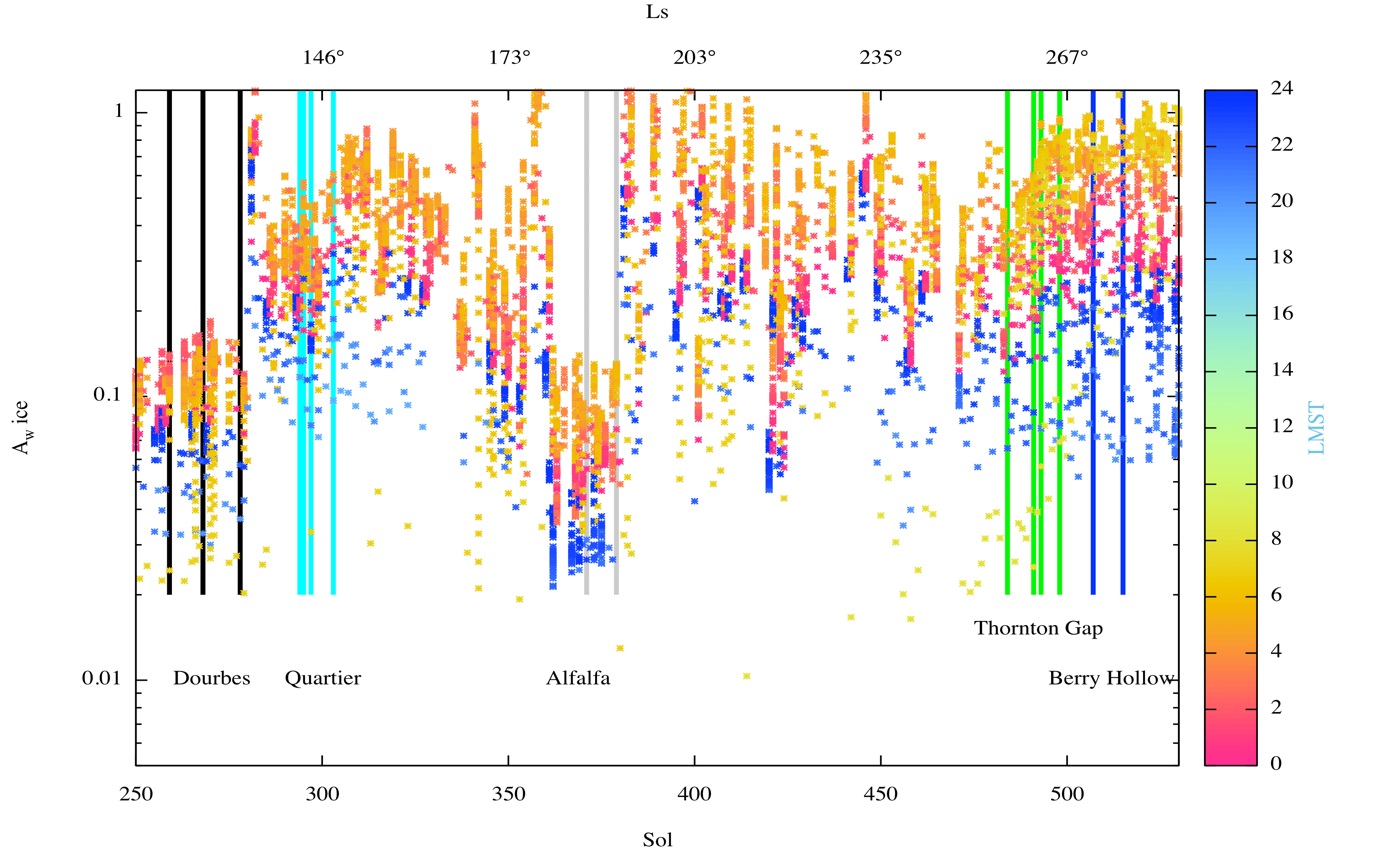


**Figure S2:** **Measurements of the ground water activity** over consecutive sols during summer, autumn, and winter. Frost conditions are met when the water activity reaches values equal to 1 or higher.

**Supporting Information C**

The sample tubes of the First (contingency) Depot, which have been dropped at Three Forks, are laying on top of a flat area covered by regolith. The sample tubes are coated in alumina (white) and titanium nitride (golden parts). These coatings can interact with the incident downwelling solar radiation during the day absorbing radiation, and at night with the atmosphere above emitting infrared radiation, resulting in local temperatures that may differ slightly from the one of the bedrock and regolith. During the next years these samples will be exposed to the diurnal cycle of the surface of Jezero crater, which according to MEDA measurements can oscillate daily between 180 K and 300 K, see Figure S3-(Top).

The downwelling irradiance (190 nm – 80 μm) during the year, inferred from MEDA data, is presented in Fig. S3 bottom. The irradiance was computed from measurements acquired by the ch190-1200 nm (top 7) channel of the Radiative and Dust Sensor (RDS) and the IR1 channel of the Thermal Infrared Sensor (TIRS), both integrated in MEDA. The top7 channel measures solar irradiance between 190 nm and 1.2 μm, presenting a particular angular response function defined by the sensor mask, which can be converted to downwards irradiance by using a radiative transfer model. Here, we used the UVISMART model to compute the downward irradiance, and also to broaden the channel spectral band to 190 nm – 5 μm. Then, the longwave downwelling irradiance from TIRS, comprised between 5 μm and 80 μm, complemented the shortwave band inferred from the RDS. The irradiance was corrected for dust deposition.

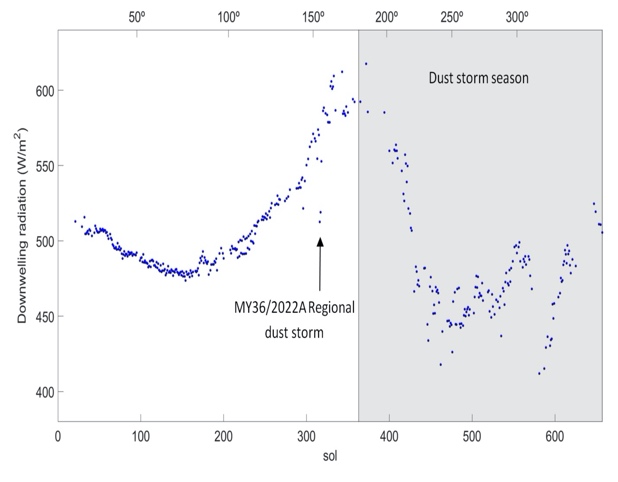


**Figure S3: (Top) Annual daily maximum, mean and minimal ground temperatures at Jezero crater as measured by MEDA. (Bottom) Diurnal maximum downwelling irradiance** during the year, inferred from MEDA data. The dust storm season (180° < Ls < 360° ) is highlighted in gray color, as well as the MY36/2022A regional dust storm, where the downwelling radiation was strongly reduced due to the large increase in atmospheric optical depth.

**Supporting Information D**

To place MEDA in-situ observations into a climatic context we include here the results of simulations done with the Mars Planetary Climate Model (PCM), developed at the Laboratoire de Météorologie Dynamique (LMD (<https://web.lmd.jussieu.fr/~lmdz/planets/mars/user_manual.pdf>) that include the dust scenario of MY36 (Montalbone et al., 2023). The water vapor VMR annual variation peaks around Ls=150°, showing significant agreement with the observations. This peak follows the release of water vapor from the north polar cap. Atmospheric flow from the summer to the winter hemisphere transports this water vapor which reaches mid-northern latitudes (and Jezero) around Ls=150°. Water ice clouds are also important atmospheric conveyors of water in the region, and they also peak at Ls=150° around Jezero. A relatively dry atmosphere prevails the rest of the year in the region. The model predicts about 500 ppm during the maximum at about 20 LMST, which is twice as much as measured at that time and season by MEDA. The model shows a factor three reduction of the VMR during the period 22:00 to 06:00 and a sudden increase during 7:00 and 8:00, although in the model this strong diurnal change is confined to only the lowest ~200 m of the atmosphere, which implies that roughly 2% of the atmosphere directly exchanges with the surface on a diurnal timescale. In this model, this is due to the condensation and sublimation of surface frost on the surface. This model does not incorporate absorption/desorption of mineral grains or hydration/dehydration but allows for water ice formation on the surface. Overall, the modelled atmosphere is wetter than the in-situ observations. This could be due to absorption/desorption processes which are not considered in the GCM, the model resolution, the altitude of the last near-surface atmospheric layer (4 m) and other errors produced by the model approximations on the water sources, cloud microphysics, etc.. The order of magnitude of the total column of water obtained from the CGM is also comparable with that calculated from MEDA measurements and shows a similar annual trend.


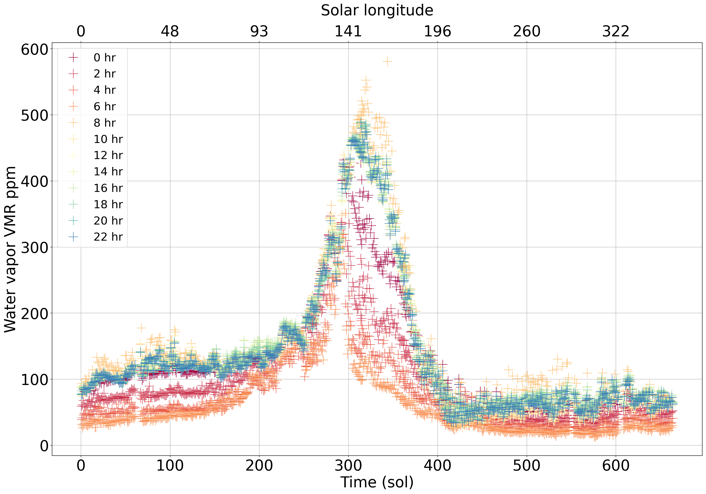

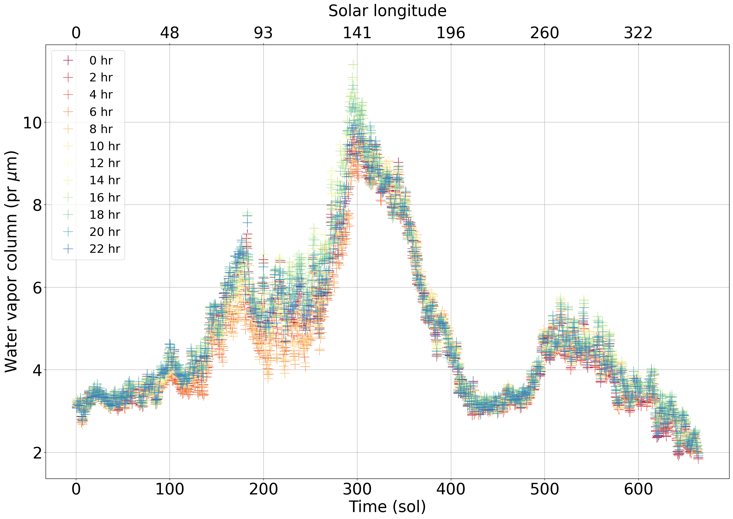

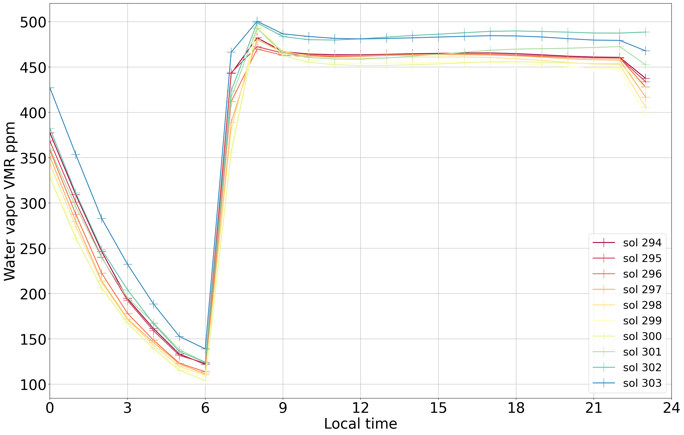


**Figure S4: Results from the LMD General Circulation Model**. (Top) The annual variability of the water VMR at 4 m above the surface, shows a comparable behaviour with the observed MEDA measurements at 1.5 m above the surface. (Center) The annual variability of the total column of water is comparable with the one measured by MEDA. (Bottom) The diurnal cycle of VMR shows a factor three reduction of the VMR during the period 22:00 to 06:00 and a sudden increase during 7:00 and 8:00.


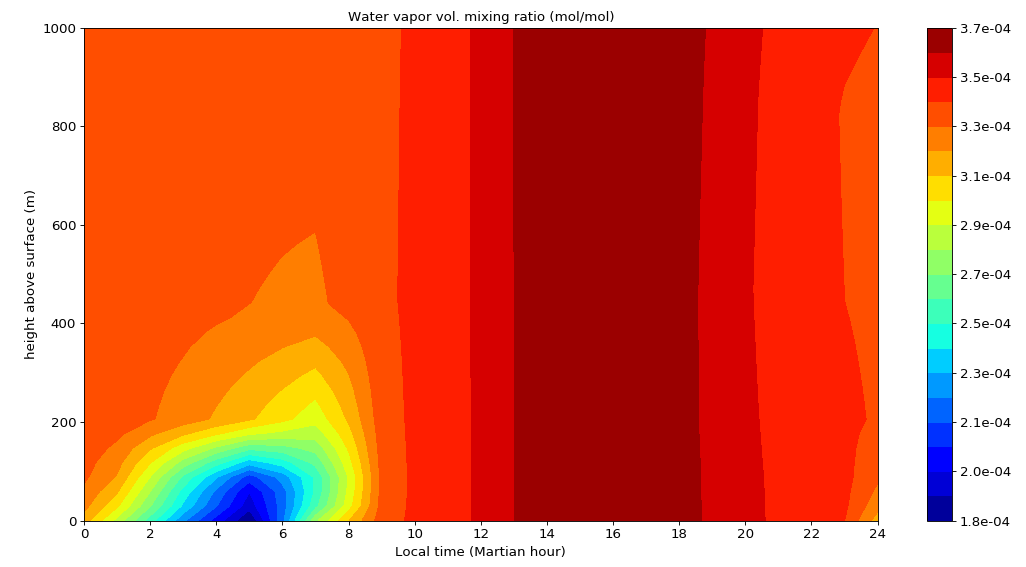


**Figure S5: Results from the Mars Climate Database showing water vapor volume mixing ratio as a function of local time and height above the surface.** In this model, the strong dependence on local time shown in the bottom panel of Figure S4 is confined to the lowest ~200 m of the atmosphere implying that ~2% of the atmosphere directly exchanges with the surface on a diurnal timescale.

When the temperature drops at night, the surface at Jezero reaches saturation and frost can be formed. This frost would be stable for several hours every day. The highest thickness of the frost layer is reached during Ls=150°, and is estimated to be of about 0.5 μm, see Figure S6. Frost sublimates during sunrise when the surface temperature increases.


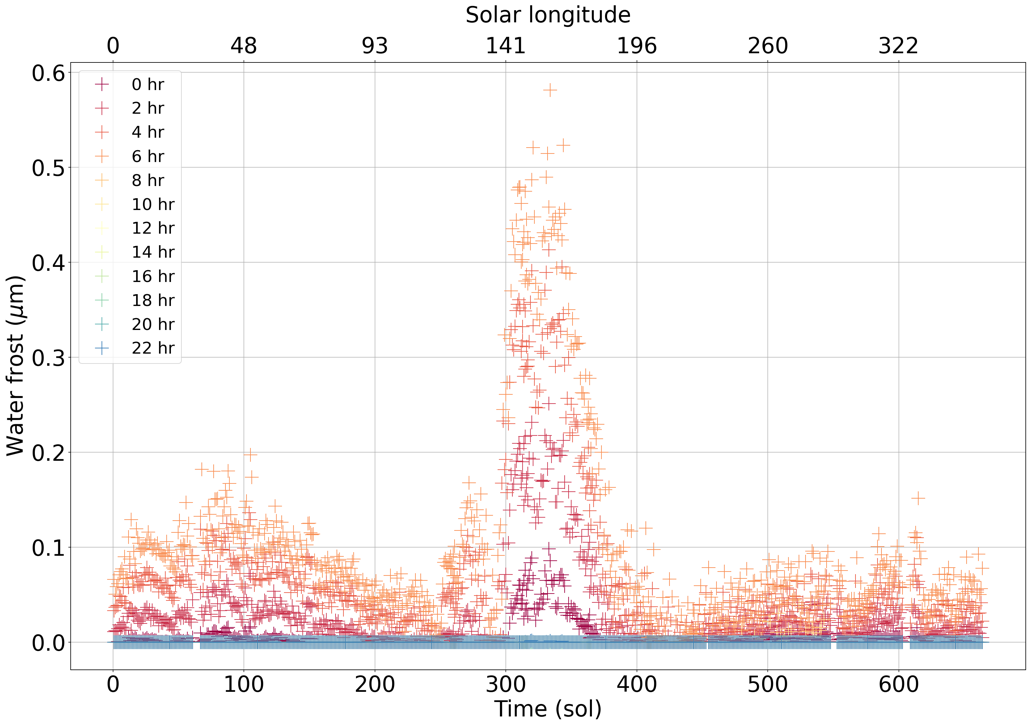

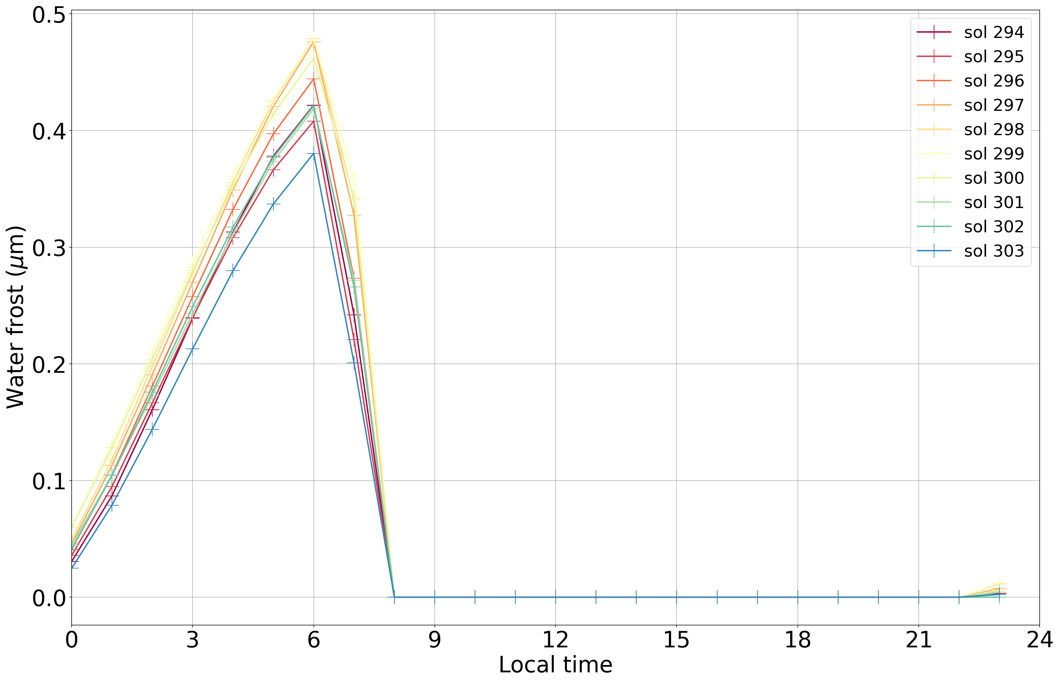


**Figure S6: Results from the LMD General Circulation Model. (Top) The annual variability of the total column of precipitated water in the form of frost**. This happens when saturation is reached on the surface. The model does not allow for absorption/desorption but includes surface frost condensation and sublimation. (Bottom) The diurnal variability of the frost thickness for the same sols shown in Figure S4. When water condenses on the surface as frost this leads to a reduction of the water VMR in the atmosphere.

**Supporting Information E**

Figure S7 shows the graph of Figure 4 divided into four seasons: the spring equinox starts at L_s_=0°, the summer solstice at L_s_=90°, the autumnal equinox at L_s_=180°, and the winter solstice at L_s_=270°.


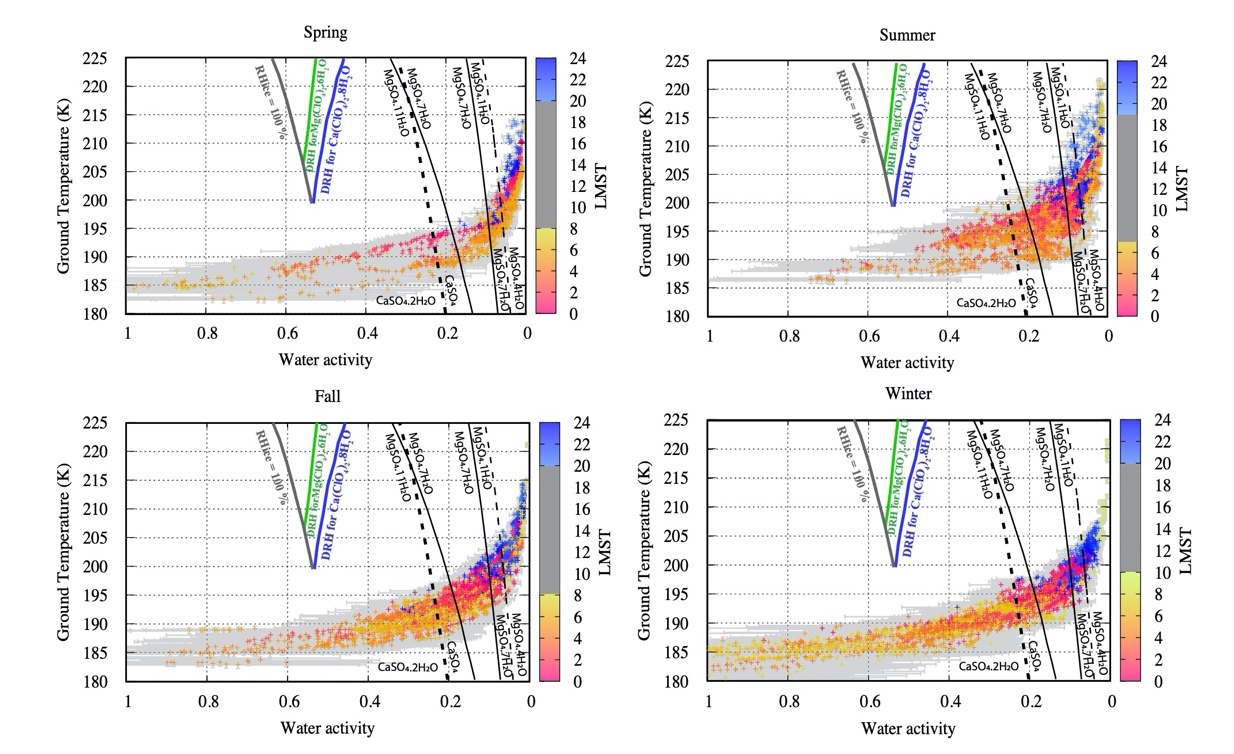


**Figure S7: Diurnal variation in LMST of the derived surface water activity and measured ground temperature provided by MEDA per season.** The environmental data are overlayed for illustration with the hydration lines of calcium and magnesium sulfates and calcium perchlorate deliquescence and efflorescence lines. Daytime conditions allow for the hydration of sulfates throughout all seasons. The water activity is derived assuming equilibrium, from the relative humidity with respect to liquid, as a_w_=RH/100, For illustration, the DRH and hydration state lines of some perchlorates and sulfate salts are included for reference (Fischer et al., 2019, Chou, I.-M and Seal II, 2007).
